# Tropical dung beetle morphological traits predict functional traits and show intraspecific differences across land uses

**DOI:** 10.1002/ece3.4218

**Published:** 2018-08-05

**Authors:** Elizabeth H. Raine, Claudia L. Gray, Darren J. Mann, Eleanor M. Slade

**Affiliations:** ^1^ Department of Zoology University of Oxford Oxford UK; ^2^ School of Life Sciences University of Sussex Brighton UK; ^3^ Oxford University Museum of Natural History Oxford UK; ^4^ Lancaster Environment Centre University of Lancaster Lancaster UK

**Keywords:** behavioral traits, Borneo, functional diversity, habitat change, oil palm, phenotypic plasticity, Scarabaeinae, tropical forest

## Abstract

Functional traits and functional diversity measures are increasingly being used to examine land use effects on biodiversity and community assembly rules. Morphological traits are often used directly as functional traits. However, behavioral characteristics are more difficult to measure. Establishing methods to derive behavioral traits from morphological measurements is necessary to facilitate their inclusion in functional diversity analyses. We collected morphometric data from over 1,700 individuals of 12 species of dung beetle to establish whether morphological measurements can be used as predictors of behavioral traits. We also compared morphology among individuals collected from different land uses (primary forest, logged forest, and oil palm plantation) to identify whether intraspecific differences in morphology vary among land use types. We show that leg and eye measurements can be used to predict dung beetle nesting behavior and period of activity and we used this information to confirm the previously unresolved nesting behavior for *Synapsis ritsemae*. We found intraspecific differences in morphological traits across different land use types. Phenotypic plasticity was found for traits associated with dispersal (wing aspect ratio and wing loading) and reproductive capacity (abdomen size). The ability to predict behavioral functional traits from morphology is useful where the behavior of individuals cannot be directly observed, especially in tropical environments where the ecology of many species is poorly understood. In addition, we provide evidence that land use change can cause phenotypic plasticity in tropical dung beetle species. Our results reinforce recent calls for intraspecific variation in traits to receive more attention within community ecology.

## 1 INTRODUCTION

Trait‐based approaches are considered essential for establishing general principles in community ecology (McGill, Enquist, Weiher, & Westoby, 2006). Functional traits describe morpho‐physio‐phenological traits which indirectly impact an organism's fitness through their effects on growth, reproduction, and survival (Violle et al., 2007). The distribution and frequency of functional traits can be used to calculate a range of indices for a sample, for example functional diversity, evenness, and dispersion (Albert et al., 2012; Mouillot, Graham, Villéger, Mason, & Bellwood, 2013). Both functional traits and associated diversity measures are increasingly being used to examine human impacts on biodiversity, and the consequences for key ecosystem functions and services (Mouillot et al., 2013; Flynn & Gogol‐Prokurat, 2009; Hamer et al. 2015). The study of functional traits is also providing insights into how communities are structured (Messier, McGill, & Lechowicz, 2010; Violle et al., 2012) and the vulnerability of communities to future disturbance (Laliberté et al., 2010).

Morphological measurements (e.g., body mass, beak size, egg size) are frequently used directly as functional traits, as morphology typically reflects an organism's interaction with the environment and the functions it performs (Bertossa, 2011; Wainwright, 1996). These morphological traits are measured on individuals caught in the field (e.g., Edwards et al., 2014; Yates & Andrew, 2011), housed in museum collections (Bregman, Sekercioglu, & Tobias, 2014; Matthews, Sheard, & Cottee‐Jones, 2015), or taken from existing literature (e.g., Edwards, Edwards, Hamer, & Davies, 2013; Flynn & Gogol‐Prokurat, 2009). In contrast, behavioral characteristics that relate to an animal's function generally need to be observed directly from living individuals and can be more difficult to obtain. Establishing methods to derive behavioral traits from morphological measurements would therefore facilitate their inclusion in functional diversity analyses (Bertossa, 2011). In turn, the inclusion of behavioral traits would then increase the utility of functional trait analyses for ecological research.

The use of mean trait values in calculations of functional diversity indices is necessary where the resources are not available to measure all individuals, but is vulnerable to biases where sample sizes are small and could overlook important within‐species variation across different treatments or habitat types (Albert, Thuiller, Yoccoz, Douzet et al., 2010; Griffiths, Louzada, Bardgett, & Barlow, 2016; Hulshof & Swenson, 2010). The latter is a particular risk for species that show high morphological plasticity, as morphological traits could vary greatly among individuals of the same species in different habitats (Albert, Thuiller, Yoccoz, Soudant et al., 2010; Petchey & Gaston, 2006). The extent and magnitude of intraspecific variation in traits needs to be understood in order to interpret the effects of land use change, the processes structuring communities, such as species coexistence (Violle et al., 2012), and the interactions between populations (Bolnick et al., 2011). Despite an increasing interest in intraspecific trait variation in ecological research (Albert et al., 2012; Griffiths et al., 2016; Violle et al., 2012), how traits vary across different land uses has not been widely examined outside of producer systems, and variability in animal traits has been largely overlooked (Albert, Thuiller, Yoccoz, Soudant et al., 2010; Albert, Grassein, Schurr, Vieilledent, & Violle, 2011 but see Moretti et al., 2017).

Dung beetles (Coleoptera: Scarabaeidae) are an ideal group in which to study variation in functional traits within and among species, as well as across different land uses. Dung beetles can be separated into distinct functional groups based on body size, period of activity (diurnal/nocturnal), and nesting behavior. They can nest within a dung pat (endocoprids or dwellers), beneath the dung (paracoprids or tunnellers), or by rolling a dung ball horizontally away from the source (telocoprids or rollers) (Hanski & Cambefort, 1991). Dung beetle species can also vary in temperature tolerance (Addo‐Bediako, Chown, & Gaston, 2000) and food resource use (Filgueiras, Liberal, Aguiar, Hernández, & Iannuzzi, 2009; Hanski & Cambefort, 1991; Holter, Scholtz, & Wardhaugh, 2002). All of these functional traits directly affect the ecosystem functions performed by dung beetles, including dung removal, bioturbation, and secondary seed dispersal (Griffiths et al., 2015; Nichols et al., 2008; Slade, Mann, Villanueva, & Lewis, 2007).

To date, dung beetle behavioral traits have been derived from direct observations of dung beetle activity. However, the natural history of many species remains incomplete particularly in the tropics (Nichols, Uriarte, Bunker, & Favila, 2013). Phylogenetic relatedness is often used to predict the behavioral ecology of a species or genus where information is missing (Barton, Gibb, Manning, Lindenmayer, & Cunningham, 2011; Bregman et al., 2014; Hunt, Bergsten, & Levkanicova, 2007; Matthews et al., 2015). However, in dung beetles, some behaviors (e.g., rolling behavior) may have evolved many times from tunneling clades (Monaghan, Inward, Hunt, & Vogler, 2007), indicating that generalizations based on phylogeny alone may be an inaccurate indication of dung beetle behavioral ecology. Dung beetle morphology is known to vary between nesting behaviors (Gregory, Gómez, Oliveira, & Nichols, 2015; Hanski & Cambefort, 1991; Inward, Davies, Pergande, Denham, & Vogler, 2011; Shafiei, Moczek, & Nijhout, 2001; Simmons & Ridsdill‐Smith, 2011) and activity period (Baird, Byrne, & Scholtz, 2010; Caveney, Scholtz, & McIntyre, 1995; Hernández, Monteiro, & Favila, 2011; McIntyre & Caveney, 1998). Therefore, morphological data could be used to predict dung beetle behavioral traits more reliably than using the behavioral traits of congeneric species.

Dung beetles have been shown to display high phenotypic plasticity in certain traits in response to resource availability (Moczek & Nijhout, 2004) and environmental change (Alves & Hernández, 2017; Emlen, 1994; Moczek, 2002; Pomfret & Knell, 2006). In particular, the morphology of males of the same species has been found to vary greatly as a result of resource quality (Moczek & Emlen, 2000; Moczek & Nijhout, 2004), and differences in horn size and shape, both within and between species, are well documented (e.g., Emlen, Marangelo, Ball, & Cunningham, 2005). Intraspecific differences in morphological traits are therefore likely to be easily detected where they occur, making dung beetles the ideal study group to assess whether land use change and associated changes in resource availability may be causing intraspecific differences in functional traits.

Here, we test whether morphological traits can be used to reliably identify the behavioral traits of dung beetle species, and whether there is intraspecific variation in these traits across different land uses. Our focus is on tropical forested landscapes, where logging and conversion to agriculture continue to cause a reduction in species richness and the abundance of many taxa, including dung beetles (Gray, Slade, Mann, & Lewis, 2014; Nichols et al., 2007; Slade, Mann, & Lewis, 2011). These changes correspond to a loss of functional diversity in dung beetles (Edwards et al., 2013, 2014; Gray et al., 2014; Senior et al., 2013) and impact ecosystem functions (Slade et al., 2011). We used measurements from dung beetle species collected in old‐growth forest, logged forest, and oil palm plantations in Sabah, Borneo, to ask the following questions:

1. Can morphological characteristics be used to separate known behavioral functional traits in dung beetles?
2. Is there intraspecific variation in dung beetle morphological traits across different tropical land uses?

## 2 METHODS

### 2.1 Data collection

Dung beetles (Coleoptera: Scarabaeinae) were sampled in Sabah, Malaysian Borneo during February and March 2011. We sampled nine sampling points at three sites in each of three land uses (*n *=* *27): old‐growth forest (OG) which had never been logged; logged forest (LF) which had experienced 2–3 periods of timber extraction, and oil palm plantations (OP) in which palms were planted between 2000 and 2006. All sampling points were located within the Stability of Altered Forest Ecosystems (S.A.F.E.) project, encompassing the old‐growth forest at Maliau Basin, (see Reynolds, Payne, Sinun, Mosigil, & Walsh, 2011; Ewers et al., 2011 for further details). The nine sampling points in each site were 178 m apart, and the three sites in each land use were 1.5 km apart. At each sampling point, we placed a pitfall trap baited with 25 g of human feces and filled with a solution of water, salt, and detergent (Gray et al., 2014). Trap contents were collected after 48 hours and stored in 75% ethanol in a freezer until beetles were identified and measurements taken.

Specimens were identified using Balthasar (1963), Boucomont (1914), the works on Bornean Scarabaeinae by Ochi and Kon (e.g., Ochi, 1996), and the reference collections housed in the Oxford University Museum of Natural History (OUMNH). Voucher collections are deposited at the OUMNH, Universiti Malaysia Sabah, and the Forest Research Centre in Sandakan, Sabah.

To ensure a minimum of 25 individuals from each species from each site were measured, we focused on 12 of the most abundant and widespread species, including at least one from each of the major functional groups. We combined the samples from all nine traps from each site. We measured as many randomly selected individuals as possible in the available time (*n* = 25–36 individuals per species per site). Species names, functional groups (as identified by Slade et al., 2007), and the number of individuals measured per species are given in Supporting information Table S1.

We were specifically interested in the species *Synapsis ritsemae* (Lansberge) as its nesting behavior is unresolved. *Synapsis ritsemae* is the only member of its genus to occur in Borneo. This genus has traditionally been classified as a nocturnal tunneller based on its taxonomic position within the Coprini (Davis, Scholtz, & Philips, 2002; Philips, Pretorius, & Scholtz, 2004), and field observations of Masumoto (1973), yet species within this genus have been recorded to perform dung ball formation for rolling as well as tunneling activity (Kon, Ochi, Kusakabe, & Araya, 2004; Zidek & Pokorny, 2010). If morphological traits alone can be reliably used to separate species according to their behaviors, we may be able to resolve the behavior of *Synapsis ritsemae* from morphological measurements. As no individuals were collected from pitfall trap contents in this study, 29 mounted specimens from earlier work at nearby locations in Sabah, Borneo (Slade et al., 2011), were measured to address this question. As samples were already dry, wing measurements could not be taken from the mounted specimens.

For each individual beetle, we measured 13 linear dimensions and one area (details given in Supporting information Table S2 and Figure S1) using a LEICA M165 stereo microscope and morphometric software Leica Application Suite (version 3.0), from which we calculated a set of six morphological traits of potential importance for ecological functioning: relative body size, abdomen size, wing aspect ratio, wing loading, eye size, and hind leg size (description of each measurement given in Table 1). Measurements were always taken on the left appendages to account for asymmetry between sides. We took measurements of tibial width across all species at approximately one‐quarter of the leg length before the base to avoid the issue of tibial wear from digging which can vary based on beetle age. For wing measurements, wing cases were opened with forceps and the left wing removed. The wing was flattened on a glass slide using a brush and water to remove creases. All morphological measurements were accurate to ±0.005 mm. The two main forms of male dung beetles (major and minor) frequently differ in size (Simmons & Ridsdill‐Smith, 2011). We therefore recorded the sex and phenotype of the beetles (female, major male, minor male).

**Table 1:** Behavioral traits and associated morphological traits, with explanation of the morphometric measurements taken

Behavioral trait	Morphological trait	Morphometric measurement	Values near unity indicate:
Activity period	Eye size (Caveney et al., 1995)	Eye Length/Body Length	A large eye relative to body length
Reproductive capacity	Abdomen size (Srygley & Chai, 1990)	Thorax Length/Abdomen Length	A large abdomen relative to thorax
Dispersal	Wing aspect ratio (Berwaerts, Van Dyck, & Aerts, 2002)	Wing Width/Wing Length	A thin, long wing
	Wing loading (Berwaerts et al., 2002)	Wing Area/Body Area (Body Area = Body Length × Thorax Width)	A large wing area relative to body area
Relative body size	Body size (Marden, 2000)	Body Length/Body Length of that species	A large body size
Nesting behavior	Hind leg size	Hind Leg Length/Body Length	A long leg relative to body length
	Hind leg robustness	Hind Leg width/Hind Leg length	A wide, short leg

## 3 ANALYSIS

### 3.1 Can morphological characteristics be used to determine behavioral functional traits?

We ran a permutational analysis of variance (PERMANOVA) to test whether morphology differed between functional groups. Data on hind leg size, hind leg robustness, eye roundness, and eye size were used as the response variables, with functional group (diurnal tunneller, nocturnal tunneller, diurnal roller, nocturnal roller, and nocturnal roller/tunneller) as the predictor. Site was used as a grouping variable to account for spatial autocorrelation. To visualize the differences between functional groups we used a principal component analysis (PCA).

### 3.2 Is there intraspecific variation in morphological traits across different tropical land uses?

We tested whether dung beetle morphological traits varied across land uses with general linear mixed models (GLMMs). We specified land use (old‐growth, logged, oil palm), species, sex (female, major male), and the two‐way interactions Land use:Species and Land use:Sex as fixed effects, and set site as a random factor. Model selection was based on p values, starting with the full model and dropping terms for which model comparison using chi‐squared tests gave *p* > 0.05. A Gaussian error distribution was appropriate for all response variables. We excluded data on minor males, as there were insufficient measurements for all species across all land uses to estimate the Land use:Sex interaction with sex as a three‐level factor (female, major male, minor male). We selected focal species that occurred in more than one land use type. However, because dung beetles show strong affinities to particular land uses, many species were not found in all three land uses. Therefore, for each morphological trait we ran separate models for the species occurring in:

Set A) Old‐growth and logged forest (*n* = 1,315 individuals measured from 11 species across six sites: *Catharsius dayacus*,* Copris sinicus*,* Microcopris doriae*,* Onthophagus obscurior*,* Onthophagus rugicollis*,* Onthophagus vulpes*,* Paragymnopleurus maurus*,* Paragymnopleurus sparsus*,* Paragymnopleurus striatus*,* Proagaderus watanabei,* and *Sisyphus thoracicus*).
Set B) Logged forest and oil palm (*n* = 408 individuals measured from four species across six sites: *Catharsius renaudpauliana*,* Onthophagus obscurior*,* Onthophagus rugicollis*, and *Proagaderus watanabei*).

As we were unable to incorporate data on minor males into the models described above, we ran a separate analysis on the ratio of major to minor males to assess whether there were differences in the relative abundances of the two phenotypes across land use types. We used only data from tunnellers, as roller species do not have male morphs that can be clearly distinguished based on morphological traits. For each site, we calculated the number of major and minor males in the random sample and used this as a two‐column response variable in a binomial generalized linear model (GLM) with a quasibinomial error structure to account for overdispersion. Land use, species, and the two‐way interaction were fixed effects. As above, we separated data into comparisons of logged forest and old‐growth forest (39 comparisons of seven species across six sites) and logged forest versus oil palm (21 comparisons of four species across six sites), and used model selection as described above.

All analyses were run using the R software (version 3.0.2, R Core Team, 2017) and the packages vegan (version 2.0‐10, (Oksanen et al., 2017) and lme4 (version 1.1‐7, Bates, Maechler, Bolker, & Walker, 2013).

## 4 RESULTS

### 4.1 Can morphological characteristics be used to determine behavioral functional traits?

**Figure 1:**
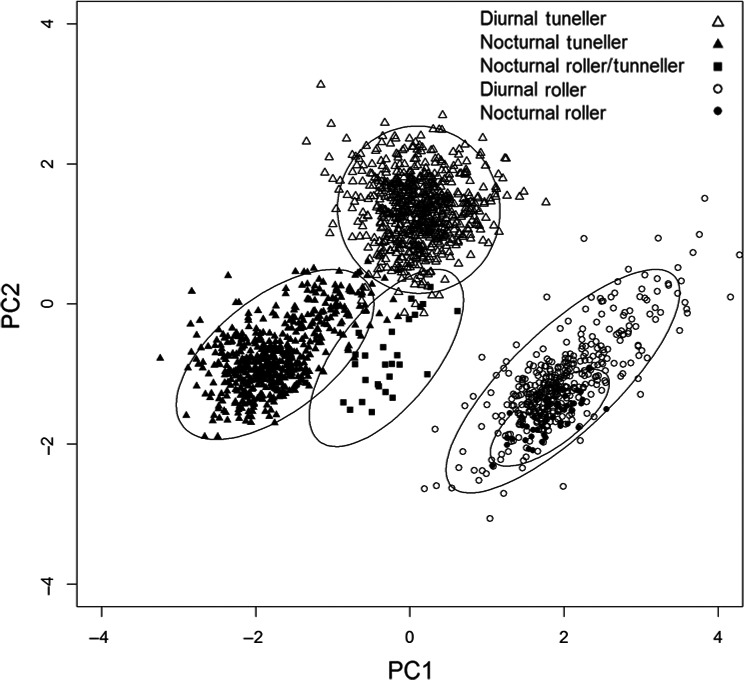
Principal component analysis of data on hind leg size, hind leg robustness, eye size, and eye roundness (see Table 2). Plot shows values for individual beetles and standard deviation of the data around the mean. The tunneller and facultative roller *Synapsis ritsemae* (■) falls between the tunneller and roller functional groups

We found statistically significant differences among the morphological traits (hind leg size, hind leg robustness, eye size, and eye roundness) of different functional groups (PERMANOVA, *F*_4,1715_ = 1182.7, *p* < 0.001). Differences between functional groups were visualized using a principal components analysis (Figure 1); PC1 explained 56.3% of the variation in the data and PC2 explained 38.6% of the total variation. More positive values of PC1 and more negative values of PC2 represent larger leg size and lower leg robustness. More positive values of both PC1 and PC2 represent rounder, smaller eyes. The tunneller and roller species separated into clusters with the rollers having greater hind leg size (relative to body size) and the tunnellers greater leg robustness (i.e., greater width for any given leg length). The nocturnal and diurnal species also separated out, with the diurnal species having rounder (i.e., greater width for any given length), smaller eyes than the nocturnal species, although this distinction was more pronounced for the tunnellers. Nocturnal rollers were represented by only one species, *Paragymnopleurus striatus*. Individuals of this species had larger, less round eyes than many of the diurnal rollers, but still fell within the overall range of values for diurnal rollers. *Synapsis ritsemae* fell clearly between the tunneller and roller clusters, but was slightly closer to the tunneller cluster. *Synapsis ritsemae* also clustered nearer the nocturnal functional groups.

### 4.2 Is there intraspecific variation in morphological traits across different tropical land uses?

**Figure 2:**
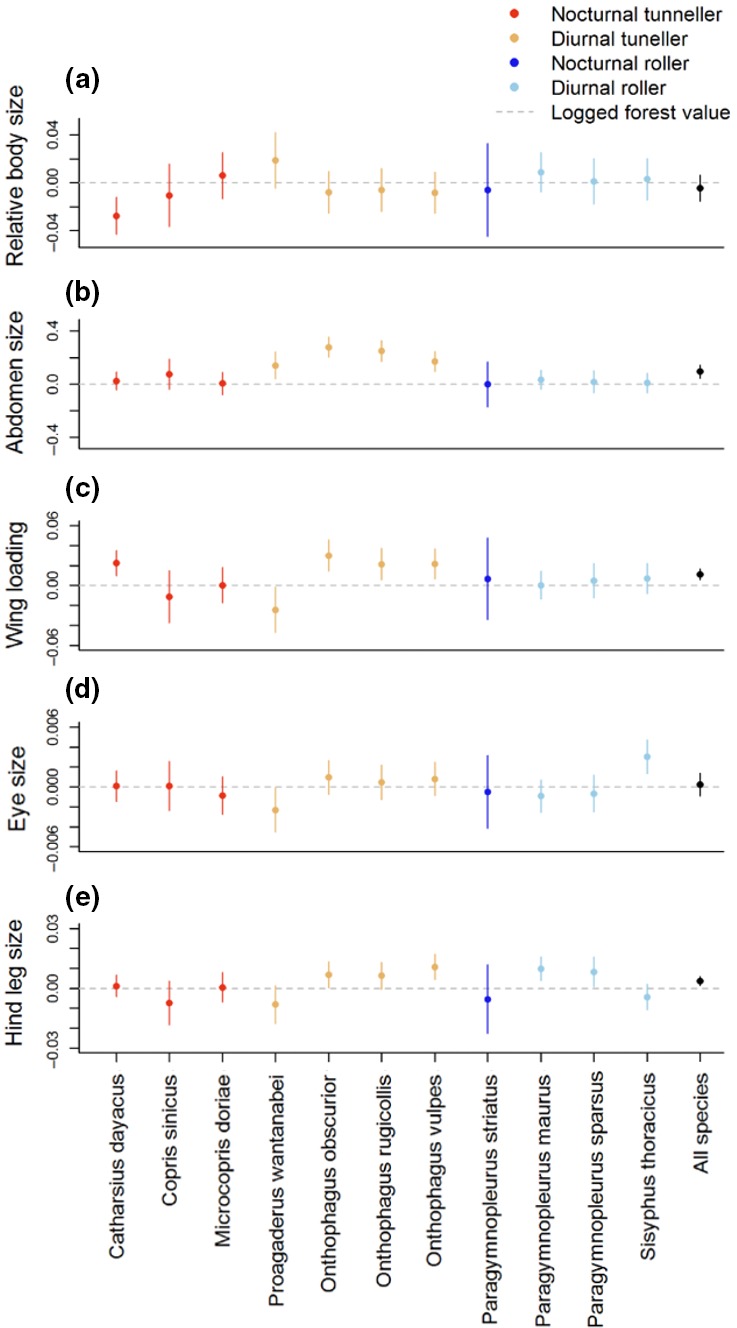
Standardized regression coefficients for morphological trait response variables for individuals in old‐growth forest relative to individuals from the same species in logged forest for each species in Set A where a significant Land use:Species interaction was observed. Responses less than zero indicate a decline the response variable moving from logged forest to old‐growth; values above zero indicate an increase. Figures show means and 95% CI obtained from model coefficients, note variation in y‐axes. Each response variable is a ratio (see Table 1) [Colour figure can be viewed at http://wileyonlinelibrary.com]

There were significant intraspecific morphological differences between dung beetles found in old‐growth and logged forest for all but one of the traits we analyzed (Table 2, Figure 2). The Land use:Species term was significant for all morphological traits apart from wing aspect ratio, suggesting that there are intraspecific differences in body size, abdomen size, wing loading, hind leg size, and eye size among land uses. For example, four species (*Catharsius dayacus* and three *Onthophagus* species) showed significantly higher wing loading in old‐growth relative to logged forest sites, and one species (*Proagaderus watanabei*) showed lower wing loading (Figure 2). The modeled mean increase in wing loading corresponds to a 6% increase in the ratio of wing area to abdomen area overall. Similarly, diurnal tunnellers that showed a change in abdomen size were found to be larger in old‐growth than that of individuals from logged forest, although the magnitude of the difference varied between species. For example, the greatest difference in abdomen size (the ratio of abdomen to thorax: higher values indicate larger abdomen, see Table 1) was shown by *Onthophagus obscurior*; the mean abdomen size of individuals was 16% larger in old‐growth compared to logged forest. Intraspecific differences in body size between old‐growth and logged forest were driven by an increase in body size of *Catharsius dayacus* in logged forest.

**Table 2:** Results of model selection based on Chi‐squared tests and *p*‐values. A separate model selection process was run for each morphological trait in each of the data sets. χ^2^, df and p‐values are given for comparisons between models with and without the term specified – following term removal. NB – in set A, *C. sinicus* has only 5 major males in old growth, and *P. striatus* only 5 individuals in total in logged forest (4 female, 1 male)

		Set A: Old growth and logged forest (11 species)			Set B: Logged forest and oil palm (4 species)		
Response	Terms	*Df*	χ^2^	*p*	*Df*	χ^2^	*p*
Relative body size	Land Use	1	0.9	0.344	1	0.02	0.898
	Sex	1	65.2	<0.001	1	35.3	<0.001
	Species	10	4450	<0.001	3	1520	<0.001
	Land Use:Species	10	29.5	<0.001	3	0.31	0.957
	Land Use:Sex	1	0.01	0.907	1	0.57	0.451
Abdomen size	Land Use	1	9.08	0.003	1	2.42	0.12
	Sex	1	120	<0.001	1	38.9	<0.001
	Species	10	2440	<0.001	3	516	<0.001
	Land Use:Species	10	98.3	<0.001	3	10.3	0.017
	Land Use:Sex	1	0.04	0.838	1	0.57	0.452
Wing aspect ratio	Land Use	1	12.1	<0.001	1	3.36	0.067
	Sex	1	10.5	0.001	1	4.62	0.032
	Species	10	2220	<0.001	3	378	<0.001
	Land Use:Species	10	15.4	0.118	3	1.32	0.724
	Land Use:Sex	1	0.58	0.445	1	0.44	0.505
Wing loading	Land Use	1	10.3	0.001	1	0.82	0.365
	Sex	1	4.79	0.029	1	7.53	0.006
	Species	10	3720	<0.001	3	849	<0.001
	Land Use:Species	10	29.9	0.001	3	7.55	0.056
	Land Use:Sex	1	0	0.987	1	0.57	0.451
Eye size	Land Use	1	0.26	0.612	1	0.13	0.719
	Sex	1	0.2	0.652	1	5.43	0.02
	Species	10	3170	<0.001	3	1130	<0.001
	Land Use:Species	10	35.1	<0.001	3	1.84	0.606
	Land Use:Sex	1	2.12	0.146	1	0.18	0.672
Hind leg size	Land Use	1	7.26	0.007	1	4.59	0.032
	Sex	1	5.78	0.016	1	1.83	0.177
	Species	10	4670	<0.001	3	325	<0.001
	Land Use:Species	10	31	0.001	3	12.1	0.007
	Land Use:Sex	1	2.26	0.132	1	0.29	0.59

For wing aspect ratio (the only response variable for which no significant Land use:Species interaction was found when comparing old‐growth and logged forest), Land Use alone was significant (Table 2). Wing aspect ratio was found to be consistently smaller for individuals of all species found in logged forest in comparison with individuals found in old‐growth. The model coefficients corresponded to a 1% decrease in the ratio of wing width to wing length from old‐growth to logged forest. The Land use:Sex interaction was not significant for any traits compared between old‐growth and logged forest, indicating that both females and major males showed similar differences across land use types. For the species found in old growth and logged forest, all morphological measurements apart from eye size differed between sexes. Males showed larger values for all measurements apart from wing loading, which was larger in females.

**Figure 3:**
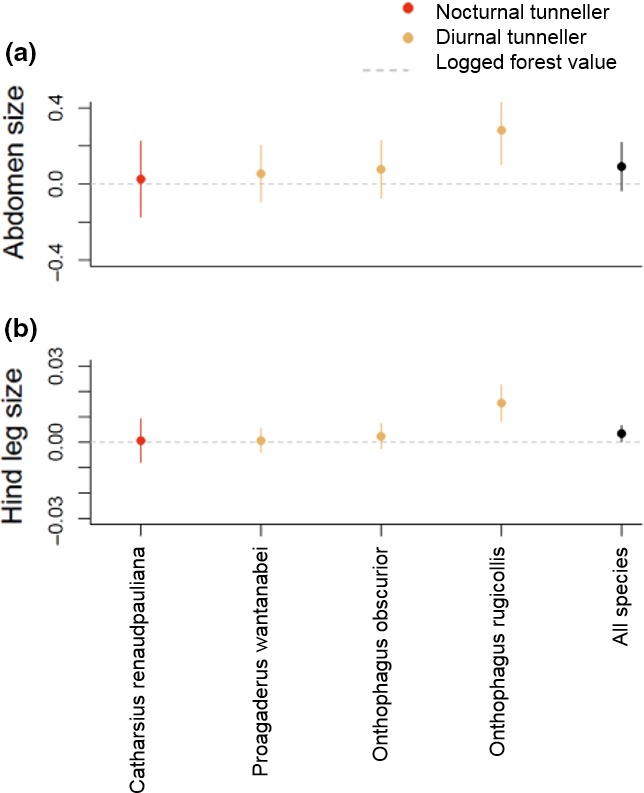
Standardized regression coefficients for morphological trait response variables for individuals in oil palm relative to individuals from the same species in logged forest for each species in Set B (logged forest:oil palm) where a significant Land use:Species interaction was observed. Responses less than zero indicate a decline the response variable moving from logged forest to oil palm; values above zero indicate an increase. Figures show means and 95% CI obtained from model coefficients, note variation in y‐axes. Each response variable is a ratio (see Table 1) [Colour figure can be viewed at http://wileyonlinelibrary.com]

Intraspecific trait differences between oil palm and logged forest only occurred in two of the six morphological traits measured: hind leg size and abdomen size (Table 2, Figure 3). For both of these traits, these results were driven by one species, the diurnal tunneller, *Onthophagus rugicollis*, that had larger hind leg size and abdomen size in oil palm compared to logged forest. The other species showed no response in relation to land use (oil palm versus logged forest). There were no significant Land use:Sex interactions; females and major males showed similar differences between logged forest and oil palm. Across all species found in logged forest and oil palm, morphological measurements differed significantly between males and females apart from hind leg size. In each case, males were larger apart from wing loading, which was larger in females.

There were three species that were found in all three land uses (old‐growth forest, logged forest, and oil palm: *Proagaderus watanabei*,* Onthophagus obscurior,* and *Onthophagus rugicollis*). By comparing Figures 2 and 3, it is evident that there were no consistent patterns of traits increasing or decreasing in magnitude across the land use gradient.

There were no statistically significant differences between the proportion of major and minor males in species found in logged forest compared to old‐growth forest (*F*_1,37_ = 1.39, *p* = 0.248), nor in species found in logged forest compared to oil palm (*F*_1,19_ = 0.16, *p* = 0.693).

## 5 DISCUSSION

Given the increasing importance of functional trait measures in community ecology, we sought to address two issues to help inform the use of morphological trait measurements. First, whether behavioral traits can be predicted from morphological measurements and second, whether there are intraspecific differences in morphological traits across land use types. Our analyses demonstrate that the functional groups of activity period and nesting behavior in dung beetles can be resolved from specific morphological measurements. We also found that for several morphological traits there are statistically significant differences among individuals of the same species from different land uses. Phenotypic plasticity was found for traits associated with dispersal (wing aspect ratio) and reproductive capacity (abdomen size).

### 5.1 Can morphological characteristics be used to determine behavioral functional traits?

Dung beetle functional groups are most often classified based on direct observation of behavior (e.g., Doube, 1990). This can be time‐consuming and a lack of data can lead to inaccuracies. It also may not be possible to observe all behavioral traits. Our results show that once behaviors and morphological measurements are known for some species, is it possible to resolve both nesting behavior and activity period (two of the most common traits used in ecological studies) for new species using morphological measurements.

Rollers and tunnellers separated along PC1, which corresponded largely to hind leg morphology. The low variance within tunneller and roller clusters suggests hind leg morphology is strongly conserved within functional groups. We were particularly interested in the position of *Synapsis ritsemae* along these axes, as this species has been classified as a tunneller (Masumoto, 1973), despite being seen to perform ball production and rolling behavior (Kon, Ochi, Kusakabe, & Araya, 2004; Zidek & Pokorny, 2010). We found that individuals of this species were clustered between the two nesting behavior guilds, albeit slightly closer to the tunneller guilds, strongly suggesting that this species is indeed capable of both behaviors. This suggests that either *Synapsis* does not belong within the Coprini or is a basal or atypical coprine in terms of its phylogeny (Tarasov & Dimitrov, 2016). This highlights how relying on phylogeny to classify functional groups may be misleading, and morphological measurements may be better predictors. Interestingly, this flexible behavior, may have evolved as a response to competition; individuals were observed to roll dung balls only when competition for dung was high, suggesting that they are facultative rollers (Slade & Mann, unpublished obs.).

Nocturnal and diurnal tunneling species separated out clearly along the PC2 axis, corresponding to eye size and roundness metrics. This confirms that for tunnellers, eye morphological characteristics can be used to determine activity period. Since *P. striatus* has been classified as a nocturnal roller, we expected that this species would form a cluster distinct from the diurnal rollers, but this was not the case. Currently, there is an incomplete understanding of the natural history of Bornean dung beetles, and species that could potentially be crepuscular such as *P. striatus* may have been misclassified as nocturnal (Slade et al., 2007). Overall, this result indicates the added value of using morphological measurements to examine and confirm behavioral traits, particularly if such behaviors are difficult to observe or are unrecorded.

The approach of classifying function through morphology could be extended to distinguish more precise, refined behavioral functional group classifications for dung beetle species (such as *Synapsis ritsemae* in this study), or to move beyond discrete classifications of behavior to metrics on a continuous scale (Fountain‐Jones 2015; Petchey & Gaston, 2006). Based on the high intraspecific variability detected in some morphological traits and the overlap between morphology and functional groups, we suggest that morphometric data be used as an additional tool to behavioral classifications of functional traits in dung beetles. Due to the lack of a resolved phylogeny for dung beetles (Monaghan et al., 2007; Tarasov & Dimitrov, 2016), it was not possible to control for the influence that phylogenetic relatedness could have on the morphology of the species studied. Despite this potential bias, the separation between functional groups is strong enough to suggest these patterns would remain if relatedness was accounted for.

### 5.2 Is there intraspecific variation in morphological traits across different tropical land uses?

Changes in dung beetle functional diversity across different land uses have been previously recorded (Andresen, 2003; Barragán, Moreno, & Escobar, 2011; Edwards et al., 2014). However, these studies have used mean trait values which do not take into account variation within species. We found significant differences in trait measurements among land use types, both within species and across all species. Griffiths et al. (2016) have recently shown intraspecific differences in neotropical dung beetle species from distinct communities in lowland tropical rainforest. Here we show that intraspecific differences are also seen with increasing human modification. It therefore seems likely that for taxa demonstrating high morphological plasticity, such as dung beetles, functional diversity measures may be incorrect if mean species trait values are used. More studies on other taxonomic groups are needed to establish the extent to which intraspecific differences in morphology occur across different land use types and the effect that including intraspecific variation has on functional diversity indices.

We found evidence of intraspecific differences between old‐growth and logged forests for several of the traits measured. Wing aspect ratio was the only trait that did not show intraspecific variation; where relatively shorter, wider wings were found for individuals of all species in logged forest in comparison with old‐growth. Abdomen size and wing loading were found to be on average larger across all species in old‐growth in comparison with logged forest. Relatively wider and shorter but smaller wings and smaller abdomens in logged forest could be indicative of a trade‐off between dispersal traits (Gibb et al., 2006) and reproductive traits (Thomas, Hill, & Lewis, 1998). This suggests traits associated to flight ability are of particular importance for dung beetle species in disturbed habitats and changes in investment between flight ability and reproductive potential could favor survival in modified land use types.

Larger bodied dung beetle species are thought to be more vulnerable to the effects of habitat disturbance, showing both reduced abundance and species richness in degraded forest (Larsen, Williams, & Kremen, 2005; Senior et al., 2013; Slade et al., 2011). However, trends seen in body size at the interspecific level are not found at the intraspecific level, as body size did not differ among land uses for most species. There was also no difference found in the relative abundance of male major/minor phenotypes across land uses. Both body size and male phenotype vary with parental investment (Moczek, 1998), which indicates that any changes in resource availability between land use types in this landscape are small, or are not being translated into reduced brood ball quality.

Hind leg size and eye size varied between species, and land use comparisons. This suggests that changes in resource availability, temperature exposure, and habitat structure do not have the same effects on all species or all traits. For example, eye size was found to be larger for some species in logged forest in comparison with old‐growth forest, and smaller for others. Thus, any possible explanation for changes in trait values affecting visibility and eyesight, such as changes in light levels through canopy structure, cannot be acting on all species in the same way. Similarly, other processes, such as competition between species, and the trade‐offs of size in any morphological dimension, appear to be acting differently on different species.

## 6 IMPLICATIONS

We have demonstrated that behavioral traits can be resolved from morphology in dung beetles, using seven morphological traits to successfully confirm or identify five behavioral traits, associated with nesting behavior, dispersal, and activity period in 12 dung beetle species. This indicates the potential to further the use of morphological characteristics in defining functional traits in dung beetles and other groups, and their use to support and supplement the classification of behavioral traits. Our findings also support recent calls for increased reporting and use of intraspecific variation in studies of morphological and functional traits (Bolnick et al., 2011; Laughlin, Joshi, Bodegom, & Bastow, 2012; Violle et al., 2012), particularly in terrestrial animal groups (see Griffiths et al., 2016). Current studies linking dung beetle communities and ecosystem functions tend to focus on community‐level attributes such as species richness, abundance, and community composition (e.g., Barnes, Emberson, Chapman, Krell, & Didham, 2014; Filgueiras, Iannuzzi, & Leal, 2011). Given that the morphological and functional traits of dung beetles affect their contribution to ecosystem function (Slade et al., 2007), biologically meaningful intraspecific differences in functionally important traits could be driving some of the changes in ecosystem function observed with habitat conversion. These subtle intraspecific effects are not yet well understood and should be incorporated into studies of how forest degradation alters ecosystem functioning.

## CONFLICT OF INTEREST

None declared.

## AUTHOR CONTRIBUTIONS

CG, DJM, and EMS conceived the study. EMS collected the samples , EHR carried out morphometric data collection and DJM assisted in species identification. CG, EHR, and EMS carried out data analysis. EHR lead the writing of the manuscript with input from all authors.

## DATA ACCESSIBILITY

Dung beetle morphometric data can be accessed the Dryad Digital Repository: https://doi.org/10.5061/dryad.1484dn3.

## References

[ece34218-bib-0001] Addo‐Bediako, A. , Chown, S. , & Gaston, K. (2000). Thermal tolerance, climatic variability and latitude. The Royal Society, 267, 739–745. 10.1098/rspb.2000.1065 PMC1690610 10819141

[ece34218-bib-0002] Albert, C. H. , de Bello, F. , Boulangeat, I. , Pellet, G. , Lavorel, S. , & Thuiller, W. (2012). On the importance of intraspecific variability for the quantification of functional diversity. Oikos, 121, 116–126. 10.1111/j.1600-0706.2011.19672.x

[ece34218-bib-0003] Albert, C. H. , Grassein, F. , Schurr, F. M. , Vieilledent, G. , & Violle, C. (2011). When and how should intraspecific variability be considered in trait‐based plant ecology? Perspectives in Plant Ecology, Evolution and Systematics, 13, 217–225. 10.1016/j.ppees.2011.04.003

[ece34218-bib-0004] Albert, C. H. , Thuiller, W. , Yoccoz, N. G. , Douzet, R. , Aubert, S. , & Lavorel, S. (2010). A multi‐trait approach reveals the structure and the relative importance of intra‐ vs. interspecific variability in plant traits. Functional Ecology, 24, 1192–1201. 10.1111/j.1365-2435.2010.01727.x

[ece34218-bib-0005] Albert, C. H. , Thuiller, W. , Yoccoz, N. G. , Soudant, A. , Boucher, F. , Saccone, P. , & Lavorel, S. (2010). Intraspecific functional variability: Extent, structure and sources of variation. Journal of Ecology, 98, 604–613. 10.1111/j.1365-2745.2010.01651.x

[ece34218-bib-0006] Alves, V. M. , & Hernández, M. I. M. (2017). Morphometric modifications in canthon quinquemaculatus castelnau 1840 (Coleoptera: Scarabaeinae): sublethal effects of transgenic maize? Insects, 8, 115 10.3390/insects8040115 PMC5746798 29065452

[ece34218-bib-0007] Andresen, E. (2003). Effect of forest fragmentation on dung beetle communities and functional consequences for plant regeneration. Ecography, 26, 87–97. 10.1034/j.1600-0587.2003.03362.x

[ece34218-bib-0008] Baird, E. , Byrne, M. , & Scholtz, C. (2010). Bearing selection in ball‐rolling dung beetles: Is it constant? Journal of Comparative Physiology A, 196, 801–806. 10.1007/s00359-010-0559-8 20635088

[ece34218-bib-0009] Barnes, A. D. , Emberson, R. M. , Chapman, H. M. , Krell, F.‐T. , & Didham, R. K. (2014). Matrix habitat restoration alters dung beetle species responses across tropical forest edges. Biological Conservation, 170, 28–37. 10.1016/j.biocon.2013.12.006

[ece34218-bib-0010] Barragán, F. , Moreno, C. , & Escobar, F. (2011). Negative impacts of human land use on dung beetle functional diversity. PLoS ONE, 6, e17976 10.1371/journal.pone.0017976 21448292 PMC3063245

[ece34218-bib-0011] Barton, P.S. , Gibb, H. , Manning, A.D. , Lindenmayer, D.B. , & Cunningham, S. A. (2011). Morphological traits as predictors of diet and microhabitat use in a diverse beetle assemblage. Biological Journal of the Linnean Society, 102, 301–310. 10.1111/j.1095-8312.2010.01580.x

[ece34218-bib-0012] Bates, D. , Maechler, M. , Bolker, B. , & Walker, S. (2013) lme4: Linear mixed‐effects models using Eigen and S4. R package versionn 1.0‐5.

[ece34218-bib-0013] Bertossa, R. C. (2011). Morphology and behavior: Functional links in development and evolution. Philosophical Transactions of the Royal Society B: Biological Sciences, 366, 2056–2068. 10.1098/rstb.2011.0035 PMC3130372 21690124

[ece34218-bib-0014] Berwaerts, K. , Van Dyck, H. , & Aerts, P. (2002). Does flight morphology relate to flight performance? An experimental test with the butterfly Pararge aegeria. Functional Ecology, 16, 484–491. 10.1046/j.1365-2435.2002.00650.x

[ece34218-bib-0015] Bolnick, D. I. , Amarasekare, P. , Araújo, M. S. , Bürger, R. , Levine, J. M. , Novak, M. , … Vasseur, D. A. (2011). Why intraspecific trait variation matters in community ecology. Trends in Ecology & Evolution, 26, 183–192. 10.1016/j.tree.2011.01.009 21367482 PMC3088364

[ece34218-bib-0016] Bregman, T. , Sekercioglu, C. , & Tobias, J. (2014). Global patterns and predictors of bird species responses to forest fragmentation: Implications for ecosystem function and conservation. Biological Conservation, 169, 372–383. 10.1016/j.biocon.2013.11.024

[ece34218-bib-0017] Caveney, S. , Scholtz, C. , & McIntyre, P. (1995). Patterns of daily flight activity in onitine dung beetles (Scarabaeinae: Onitini). Oecologia, 103, 444–452. 10.1007/BF00328682 28306992

[ece34218-bib-0018] Davis, A. , Scholtz, C. , & Philips, T. (2002). Historical biogeography of scarabaeine dung beetles. Journal of Biogeography, 29, 1217–1256. 10.1046/j.1365-2699.2002.00776.x

[ece34218-bib-0019] Doube, B. (1990). A functional classification for analysis of the structure of dung beetle assemblages. Ecological Entomology, 15, 371–383. 10.1111/j.1365-2311.1990.tb00820.x

[ece34218-bib-0020] Edwards, F. A. , Edwards, D. P. , Hamer, K. C. , & Davies, R. G. (2013). Impacts of logging and conversion of rainforest to oil palm on the functional diversity of birds in Sundaland (ed L Lens). Ibis, 155, 313–326. 10.1111/ibi.12027

[ece34218-bib-0021] Edwards, F. A. , Edwards, D. P. , Larsen, T. H. , Hsu, W. W. , Benedick, S. , Chung, A. , … Hamer, K. C. (2014). Does logging and forest conversion to oil palm agriculture alter functional diversity in a biodiversity hotspot? Animal Conservation, 17, 163–173. 10.1111/acv.12074 25821399 PMC4372061

[ece34218-bib-0022] Emlen, D. (1994). Environmental control of horn length dimorphism in the beetle Onthophagus acuminatus (Coleoptera: Scarabaeidae). Proceedings of the Royal Society of London, 256, 131–136. 10.1098/rspb.1994.0060

[ece34218-bib-0023] Emlen, D. J. , Marangelo, J. , Ball, B. , & Cunningham, C. W. (2005). Diversity in the weapons of sexual selection: Horn evolution in the beetle genus onthophagus (Coleoptera: Scarabaeidae). Evolution, 59, 1060–1084. 10.1111/j.0014-3820.2005.tb01044.x 16136805

[ece34218-bib-0024] Ewers, R. M. , Didham, R. K. , Fahrig, L. , Ferraz, G. , Hector, A. , Holt, R. D. , … Turner, E. C. (2011). A large‐scale forest fragmentation experiment: The stability of altered forest ecosystems project. Philosophical Transactions of the Royal Society B: Biological Sciences, 366, 3292–3302. 10.1098/rstb.2011.0049 PMC3179633 22006969

[ece34218-bib-0025] Filgueiras, B. K. C. , Iannuzzi, L. , & Leal, I. R. (2011). Habitat fragmentation alters the structure of dung beetle communities in the Atlantic Forest. Biological Conservation, 144, 362–369. 10.1016/j.biocon.2010.09.013

[ece34218-bib-0026] Filgueiras, B. K. C. , Liberal, C. N. , Aguiar, C. D. M. , Hernández, M. I. M. , & Iannuzzi, L. (2009). Attractivity of omnivore, carnivore and herbivore mammalian dung to Scarabaeinae (Coleoptera, Scarabaeidae) in a tropical Atlantic rainforest remnant. Revista Brasileira de Entomologia, 53, 422–427. 10.1590/S0085-56262009000300017

[ece34218-bib-0027] Flynn, D. , & Gogol‐Prokurat, M. (2009). Loss of functional diversity under land use intensification across multiple taxa. Ecology Letters, 12, 22–33. 10.1111/j.1461-0248.2008.01255.x 19087109

[ece34218-bib-0028] Fountain‐Jones . (2015) Moving beyond the guild concept : Developing a practical functional trait framework. Ecological Entomology, 40, 1–13.

[ece34218-bib-0029] Gibb, H. , Hjältén, J. , Ball, J. P. , Pettersson, R. B. , Landin, J. , Alvini, O. , & Danell, K. (2006). Wing loading and habitat selection in forest beetles: Are red‐listed species poorer dispersers or more habitat‐specific than common congenerics? Biological Conservation, 132, 250–260. 10.1016/j.biocon.2006.04.017

[ece34218-bib-0030] Gray, C. L. , Slade, E. M. , Mann, D. J. , & Lewis, O. T. (2014). Do riparian reserves support dung beetle biodiversity and ecosystem services in oil palm‐dominated tropical landscapes? Ecology and Evolution, 4, 1049–1060. 10.1002/ece3.1003 24772282 PMC3997321

[ece34218-bib-0031] Gregory, N. , Gómez, A. , Oliveira, T. M. , & Nichols, E. (2015). Big dung beetles dig deeper: Trait‐based consequences for faecal parasite transmission. International Journal for Parasitology, 45, 101–105. 10.1016/j.ijpara.2014.10.006 25496914

[ece34218-bib-0032] Griffiths, H. M. , Louzada, J. , Bardgett, R. D. , & Barlow, J. (2016). Assessing the importance of intraspecific variability in dung beetle functional traits. PLoS ONE, 11, 1–14. 10.1371/journal.pone.0145598 PMC4777568 26939121

[ece34218-bib-0033] Griffiths, H. M. H. , Louzada, J. , Bardgett, R. R. D. , Beiroz, W. , França, F. , Tregidgo, D. , & Barlow, J. (2015). Biodiversity and environmental context predict dung beetle mediated seed dispersal in a tropical forest field experiment. Ecology, 96, 1607–1619. 10.1890/14-1211.1

[ece34218-bib-0034] Hanski, I. , & Cambefort, Y. (1991). Dung beetle ecology. Princeton, NJ: Princeton University Press Princeton 10.1515/9781400862092

[ece34218-bib-0500] Hamer, K. C. , Newton, R. J. , Edwards, F. A. , Benedick, S. , Bottrell, S. H. , & Edwards, D. P. (2015). Impacts of selective logging on insectivorous birds in Borneo: the importance of trophic position, body size and foraging height. Biological Conservation, 188, 82–88. 10.1016/j.biocon.2014.09.026

[ece34218-bib-0035] Hernández, M. I. M. , Monteiro, L. R. , & Favila, M. E. (2011). The role of body size and shape in understanding competitive interactions within a community of neotropical dung beetles the role of body size and shape in understanding competitive interactions within a community of Neotropical dung beetles. Journal of Insect Science, 11, 13. 21526928 10.1673/031.011.0113 PMC3281377

[ece34218-bib-0036] Holter, P. , Scholtz, C. H. , & Wardhaugh, K. G. (2002). Dung feeding in adult scarabaeines (tunnellers and endocoprids): Even large dung beetles eat small particles. Ecological Entomology, 27, 169–176. 10.1046/j.1365-2311.2002.00399.x

[ece34218-bib-0037] Hulshof, C. M. , & Swenson, N. G. (2010). Variation in leaf functional trait values within and across individuals and species: An example from a Costa Rican dry forest. Functional Ecology, 24, 217–223. 10.1111/j.1365-2435.2009.01614.x

[ece34218-bib-0038] Hunt, T. , Bergsten, J. , & Levkanicova, Z. (2007). A comprehensive phylogeny of beetles reveals the evolutionary origins of a superradiation. Science, 318, 1913–1916. 10.1126/science.1146954 18096805

[ece34218-bib-0039] Inward, D. J. G. , Davies, R. G. , Pergande, C. , Denham, A. J. , & Vogler, A. P. (2011). Local and regional ecological morphology of dung beetle assemblages across four biogeographic regions. Journal of Biogeography, 38, 1668–1682. 10.1111/j.1365-2699.2011.02509.x

[ece34218-bib-0040] Kon, M. , Ochi, T. , Kusakabe, Y. , & Araya, K. (2004). A dung ball likely made by a male of Synapsis tridens (Coleoptera, Scarabaeidae). Kogane, 5, 13–15.

[ece34218-bib-0041] Laliberté, E. , Wells, J. A. , DeClerck, F. , Metcalfe, D. J. , Catterall, C. P. , Queiroz, C. , … Mayfield, M. M. (2010). Land‐use intensification reduces functional redundancy and response diversity in plant communities. Ecology Letters, 13, 76–86. 10.1111/j.1461-0248.2009.01403.x 19917052

[ece34218-bib-0042] Larsen, T. H. T. , Williams, N. M. N. , & Kremen, C. (2005). Extinction order and altered community structure rapidly disrupt ecosystem functioning. Ecology Letters, 8, 538–547. 10.1111/j.1461-0248.2005.00749.x 21352458

[ece34218-bib-0043] Laughlin, D. , Joshi, C. , Bodegom, P. M. , Bastow, Z. A. , & Fulé, P. Z. (2012). A predictive model of community assembly that incorporates intraspecific trait variation. Ecology letters, 15(11), 1291–1299. 22906233 10.1111/j.1461-0248.2012.01852.x

[ece34218-bib-0501] Marden, J. H. (2000). Variability in the size, composition, and function of insect flight muscles. Annual Review of Physiology, 62(1), 157–178. 10.1146/annurev.physiol.62.1.157 10845088

[ece34218-bib-0044] Masumoto, K. (1973). Observation of the Nidification of Synapsis davidi FAIRMAIRE. Entomological Review of Japan, 25, 60–62.

[ece34218-bib-0045] Matthews, T. , Sheard, C. , & Cottee‐Jones, H. (2015). Ecological traits reveal functional nestedness of bird communities in habitat islands: A global survey. Oikos, 124, 817–826. 10.1111/oik.02370

[ece34218-bib-0046] McGill, B. J. , Enquist, B. J. , Weiher, E. , & Westoby, M. (2006). Rebuilding community ecology from functional traits. Trends in Ecology and Evolution, 21, 178–185. 10.1016/j.tree.2006.02.002 16701083

[ece34218-bib-0047] McIntyre, P. , & Caveney, S. (1998). Superposition optics and the time of flight in onitine dung beetles. Journal of Comparative Physiology A, 183, 45–60. 10.1007/s003590050233

[ece34218-bib-0048] Messier, J. , McGill, B. J. , & Lechowicz, M. J. (2010). How do traits vary across ecological scales? A case for trait‐based ecology. Ecology Letters, 13, 838–848. 10.1111/j.1461-0248.2010.01476.x 20482582

[ece34218-bib-0049] Moczek, A. (1998). Horn polyphenism in the beetle Onthophagus taurus: Larval diet quality and plasticity in parental investment determine adult body size and male horn morphology. Behavioral Ecology, 9, 636–641. 10.1093/beheco/9.6.636

[ece34218-bib-0050] Moczek, A. (2002). Allometric plasticity in a polyphenic beetle. Ecological Entomology, 27, 58–67. 10.1046/j.0307-6946.2001.00385.x

[ece34218-bib-0051] Moczek, A. P. , & Emlen, D. J. (2000). Male horn dimorphism in the scarab beetle, Onthophagus taurus: Do alternative reproductive tactics favour alternative phenotypes? Animal Behavior, 59, 459–466. 10.1006/anbe.1999.1342 10675268

[ece34218-bib-0052] Moczek, A. , & Nijhout, H. (2004). Trade‐offs during the development of primary and secondary sexual traits in a horned beetle. The American Naturalist, 163, 184–191. 10.1086/381741 14970921

[ece34218-bib-0053] Monaghan, M. T. , Inward, D. J. G. , Hunt, T. , & Vogler, A. P. (2007). A molecular phylogenetic analysis of the Scarabaeinae (dung beetles). Molecular Phylogenetics and Evolution, 45, 674–692. 10.1016/j.ympev.2007.06.009 17656114

[ece34218-bib-0054] Moretti, M. , Dias, A. T. , Bello, F. , Altermatt, F. , Chown, S. L. , Azcárate, F. M. , … Ibanez, S. (2017). Handbook of protocols for standardized measurement of terrestrial invertebrate functional traits. Functional Ecology, 31(3), 558–567.

[ece34218-bib-0055] Mouillot, D. , Graham, N. A. , Villéger, S. , Mason, N. W. H. , & Bellwood, D. R. (2013). A functional approach reveals community responses to disturbances. Trends in Ecology and Evolution, 28, 167–177. 10.1016/j.tree.2012.10.004 23141923

[ece34218-bib-0056] Nichols, E. , Larsen, T. , Spector, S. , Davis, A. L. , Escobar, F. , Favila, M. , & Vulinec, K. (2007). Global dung beetle response to tropical forest modification and fragmentation: A quantitative literature review and meta‐analysis. Biological Conservation, 137, 1–19. 10.1016/j.biocon.2007.01.023

[ece34218-bib-0057] Nichols, E. , Spector, S. , Louzada, J. , Larsen, T. , Amezquita, S. , & Favila, M. E. (2008). Ecological functions and ecosystem services provided by Scarabaeinae dung beetles. Biological Conservation, 141, 1461–1474. 10.1016/j.biocon.2008.04.011

[ece34218-bib-0058] Nichols, E. , Uriarte, M. , Bunker, D. , & Favila, M. (2013). Trait‐dependent response of dung beetle populations to tropical forest conversion at local and regional scales. Ecology, 94, 180–189. 10.1890/12-0251.1 23600252

[ece34218-bib-0059] Ochi, T. (1996). Studies on the family Scarabaeidae (Coleoptera) from Borneo. I. Identification keys to subfamilies, tribes and genera. Giomale Italia Entomologia, 8, 37–54.

[ece34218-bib-0060] Oksanen, J. , Kindt, R. , Legendre, P. , O'hara, B. , Stevens, M.H.H. , Oksaken, M. , & Suggests, M . (2017) The vegan package. Community ecology package 10.

[ece34218-bib-0061] Petchey, O. L. , & Gaston, K. J. (2006). Functional diversity: Back to basics and looking forward. Ecology Letters, 9, 741–758. 10.1111/j.1461-0248.2006.00924.x 16706917

[ece34218-bib-0062] Philips, T. , Pretorius, E. , & Scholtz, C. (2004). A phylogenetic analysis of dung beetles (Scarabaeinae: Scarabaeidae): Unrolling an evolutionary history. Invertebrate Systematics, 18, 53–88. 10.1071/IS03030

[ece34218-bib-0063] Pomfret, J. , & Knell, R. (2006). Sexual selection and horn allometry in the dung beetle Euoniticellus intermedius. Animal Behavior, 71, 565–576.

[ece34218-bib-0064] R Core Team . (2017) R: A Language and Environment for Statistical Computing.

[ece34218-bib-0065] Reynolds, G. , Payne, J. , Sinun, W. , Mosigil, G. , & Walsh, R. P. D. (2011). Changes in forest land use and management in Sabah, Malaysian Borneo, 1990‐2010, with a focus on the Danum Valley region. Philosophical Transactions of the Royal Society B: Biological Sciences, 366, 3168–3176. 10.1098/rstb.2011.0154 PMC3179641 22006960

[ece34218-bib-0066] Senior, M. J. M. , Hamer, K. C. , Bottrell, S. , Edwards, D. P. , Fayle, T. M. , Lucey, J. M. , … Stewart, C. (2013). Trait‐dependent declines of species following conversion of rain forest to oil palm plantations. Biodiversity and Conservation, 22, 253–268.

[ece34218-bib-0067] Shafiei, M. , Moczek, A. , & Nijhout, H. (2001). Food availability controls the onset of metamorphosis in the dung beetle Onthophagus taurus (Coleoptera: Scarabaeidae). Physiological Entomology, 26, 173–180. 10.1046/j.1365-3032.2001.00231.x

[ece34218-bib-0068] Simmons, L. , & Ridsdill‐Smith, T. (2011). Ecology and evolution of dung beetles. London, UK: John Wiley & Sons 10.1002/9781444342000

[ece34218-bib-0069] Slade, E. M. , Mann, D. J. , & Lewis, O. T. (2011). Biodiversity and ecosystem function of tropical forest dung beetles under contrasting logging regimes. Biological Conservation, 144, 166–174. 10.1016/j.biocon.2010.08.011

[ece34218-bib-0070] Slade, E. M. , Mann, D. J. , Villanueva, J. F. , & Lewis, O. T. (2007). Experimental evidence for the effects of dung beetle functional group richness and composition on ecosystem function in a tropical forest. The Journal of Animal Ecology, 76, 1094–1104. 10.1111/j.1365-2656.2007.01296.x 17922706

[ece34218-bib-0071] Srygley, R. , & Chai, P. (1990). Flight morphology of Neotropical butterflies: Palatability and distribution of mass to the thorax and abdomen. Oecologia, 84, 491–499. 10.1007/BF00328165 28312965

[ece34218-bib-0072] Tarasov, S. , & Dimitrov, D. (2016). Multigene phylogenetic analysis redefines dung beetles relationships and classification (Coleoptera: Scarabaeidae: Scarabaeinae). BMC Evolutionary Biology, 16, 257 10.1186/s12862-016-0822-x 27899070 PMC5129633

[ece34218-bib-0073] Thomas, C. D. , Hill, J. K. , & Lewis, O. T. (1998). Evolutionary consequences of habitat fragmentation in a localized butterfly. Journal of Animal Ecology, 67, 485–497. 10.1046/j.1365-2656.1998.00213.x

[ece34218-bib-0074] Violle, C. , Enquist, B. J. , McGill, B. J. , Jiang, L. I. N. , Albert, C. H. , Hulshof, C. , … Messier, J. (2012). The return of the variance: intraspecific variability in community ecology. Trends in ecology & evolution, 27(4), 244–252. 22244797 10.1016/j.tree.2011.11.014

[ece34218-bib-0075] Violle, C. , Navas, M. L. , Vile, D. , Kazakou, E. , Fortunel, C. , Hummel, I. , & Garnier, E. (2007). Let the concept of trait be functional!. Oikos, 116, 882–892. 10.1111/j.0030-1299.2007.15559.x

[ece34218-bib-0076] Wainwright, P. (1996). Ecological explanation through functional morphology: The feeding biology of sunfishes. Ecology, 77, 1336–1343. 10.2307/2265531

[ece34218-bib-0077] Yates, M. , & Andrew, N. (2011). Comparison of ant community composition across different land‐use types: Assessing morphological traits with more common methods. Australian Journal of Entomology, 50, 118–124. 10.1111/j.1440-6055.2010.00795.x

[ece34218-bib-0078] Zidek, J. , & Pokorny, S. (2010). Review of Synapsis Bates (Scaraaeidae: Scaravaeine: Coprini) with description of a new species. Insecta Mundi, 142, 1–21.

