# Supplementary figures and images for: Tropical dung beetle morphological traits predict functional traits and show intraspecific differences across land uses
Source: Ecol Evol. 2018 Aug 5;8(17):8686–96. doi: 10.1002/ece3.4218 (PMC6157683; doi:10.1002/ece3.4218)

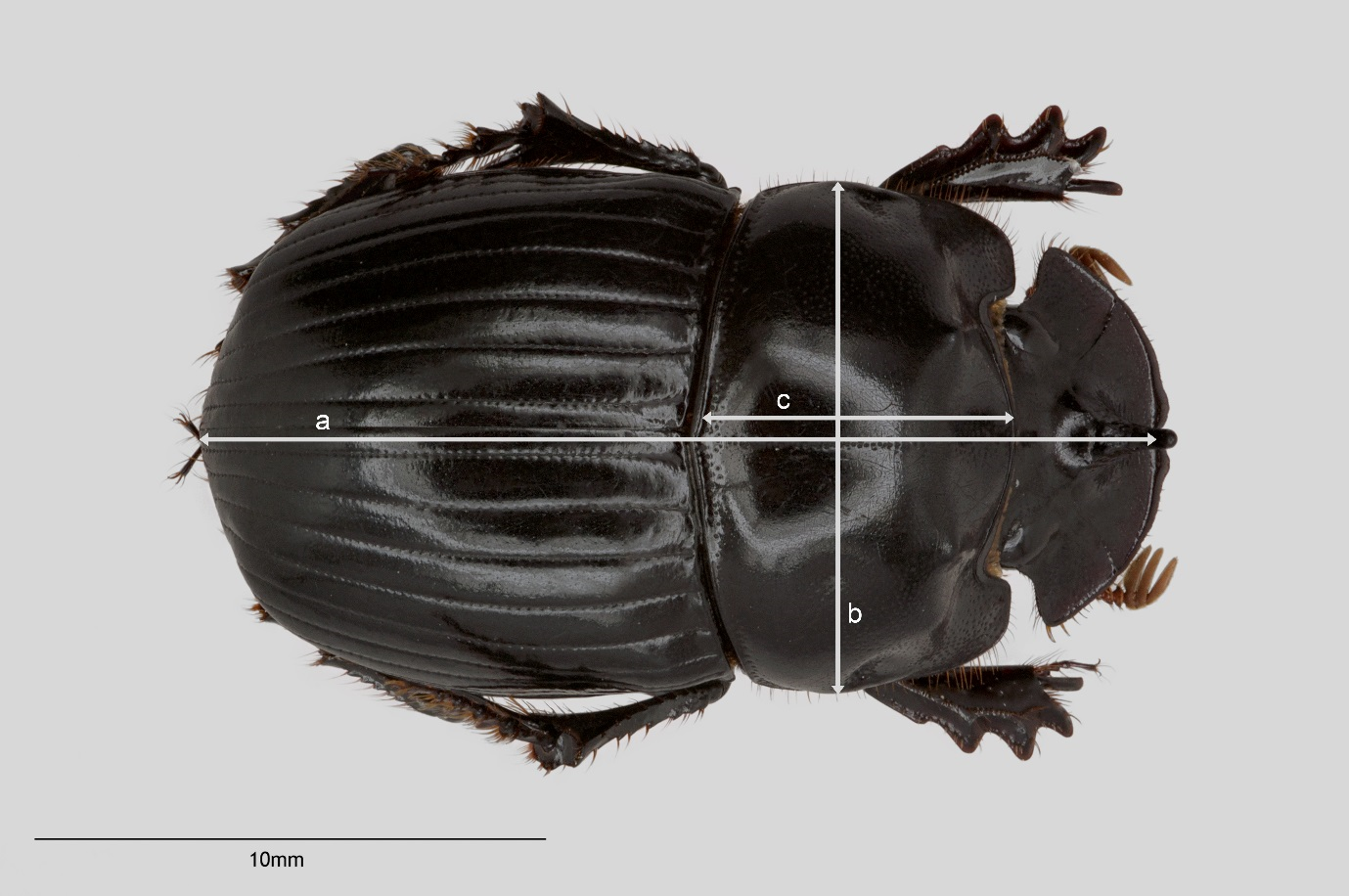

Supplement: Supplementary file 1 [file ECE3-8-8686-s001.tif]
