# Supplementary material for: Tropical dung beetle morphological traits predict functional traits and show intraspecific differences across land uses
Source: Ecol Evol. 2018 Aug 5;8(17):8686–96. doi: 10.1002/ece3.4218 (PMC6157683; doi:10.1002/ece3.4218)
Supplement: Supplementary file 2 [file ECE3-8-8686-s002.docx]

# Online supporting Information

## **Table S1**

Numbers of individuals of each species measured and their functional group (taken from Slade *et al.*, 2011)

| **Species** | **Functional group**  (From Slade *et al.,* 2011) | **Total number of individuals**  **measured** |
| --- | --- | --- |
| *Catharsius dayacus* Hope | Large nocturnal tunneller | **296** |
| *Catharsius renaudpauliani* Ochi & Kon | Large nocturnal tunneller | **88** |
| *Copris sinicus* Hope | Large nocturnal tunneller | **50** |
| *Microcopris doriae* Harold | Small nocturnal tunneller | **112** |
| *Onthophagus obscurior* Boucomont | Small diurnal tunneller | **220** |
| *Onthophagus rugicollis* Harold | Small diurnal tunneller | **155** |
| *Onthophagus vulpes* Harold | Small diurnal tunneller | **155** |
| *Paragymnopleurus maurus* Sharp | Large diurnal roller | **157** |
| *Paragymnopleurus sparsus* Sharp | Large diurnal roller | **109** |
| *Paragymnopleurus striatus* Sharp | Large nocturnal roller | **46** |
| *Proagoderus wantanabei* Ochi & Kon | Large diurnal tunneller | **165** |
| *Sisyphus thoracicus* Sharp | Small diurnal roller | **138** |
| *Synapsis ritsemae* Lansberge | Unresolved | **29** |
| **Total** | | **1716** |

# **Table S2.**

Description of morphological measurements taken. Letters correspond to details in Figure S1.

| **Measurement** | **Description** |
| --- | --- |
| Body Length (a) | Total length of beetle from tip of head to base of wing case (mm) |
| Thorax width (b) | Maximum dorsal width of pronotum disk (mm) |
| Thorax length (c) | Dorsal length of centre of pronotum disk (mm) |
| Head Width (d) | Maximum dorsal width of head (mm) *(not used in analysis)* |
| Eye length (e) | Maximum length of eye (mm) |
| Eye width (f) | Maximum width of eye (mm) |
| Head Length (g) | Dorsal length of centre of head (mm) *(not used in analysis)* |
| Wing Length (h) | Maximum length of wing (mm) |
| Wing Width (i) | Maximum width of wing (mm) |
| Wing Area (j) | Area of wing (mm²) |
| Abdomen length (k, n) | Maximum ventral length of abdomen (mm) |
| Tibia Length (l,p) | Maximum length of tibia, excluding tibial spurs (mm) |
| Tibia Width (m,o) | Width of tibia one third from base (mm) |


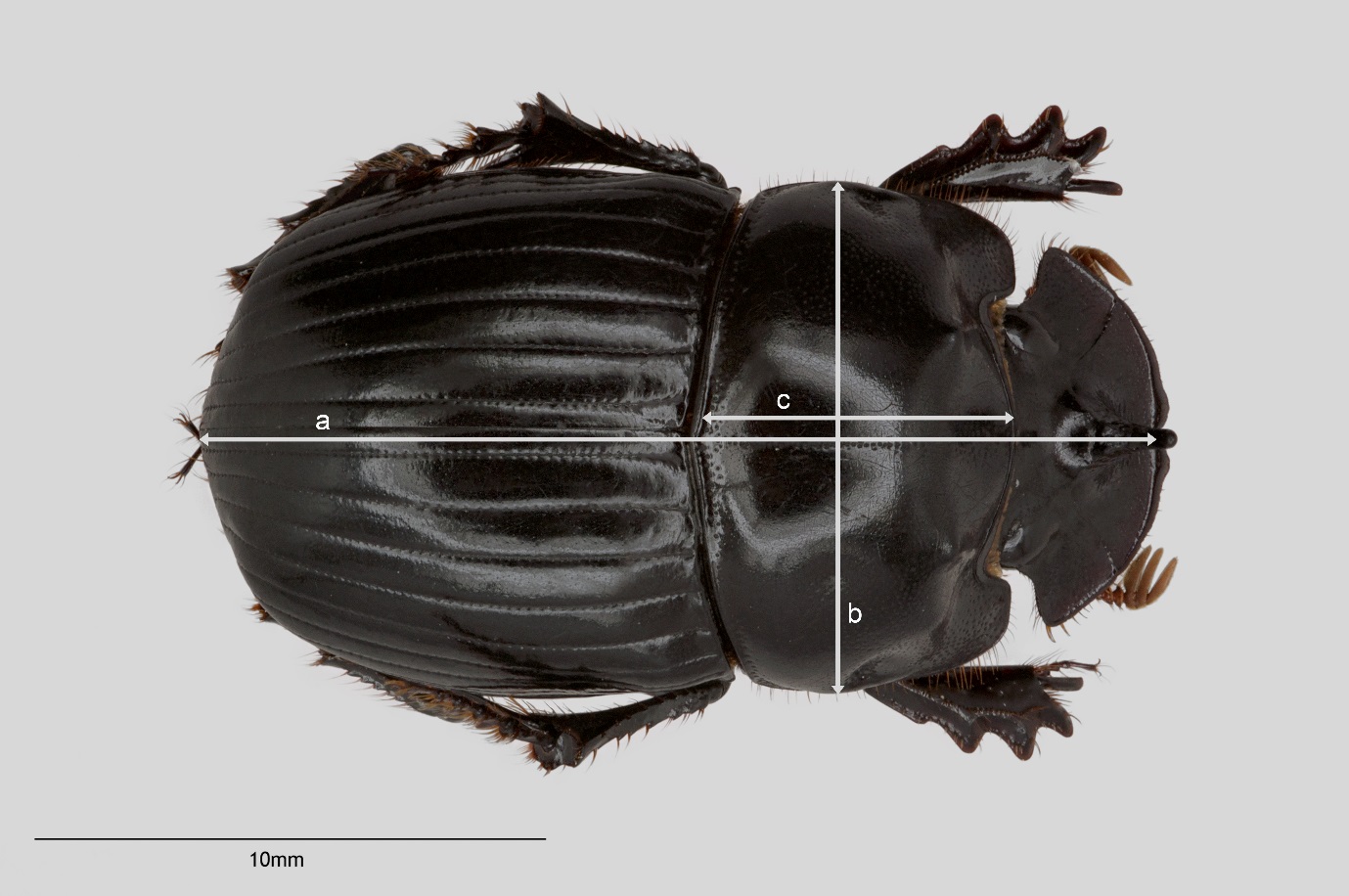


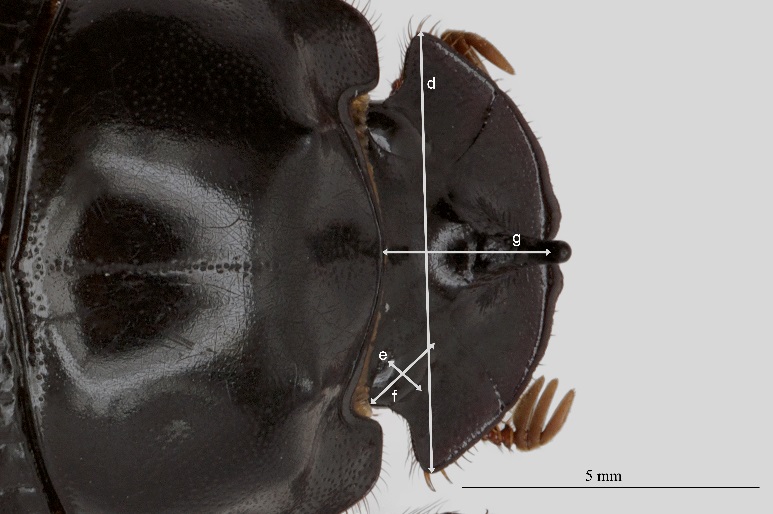


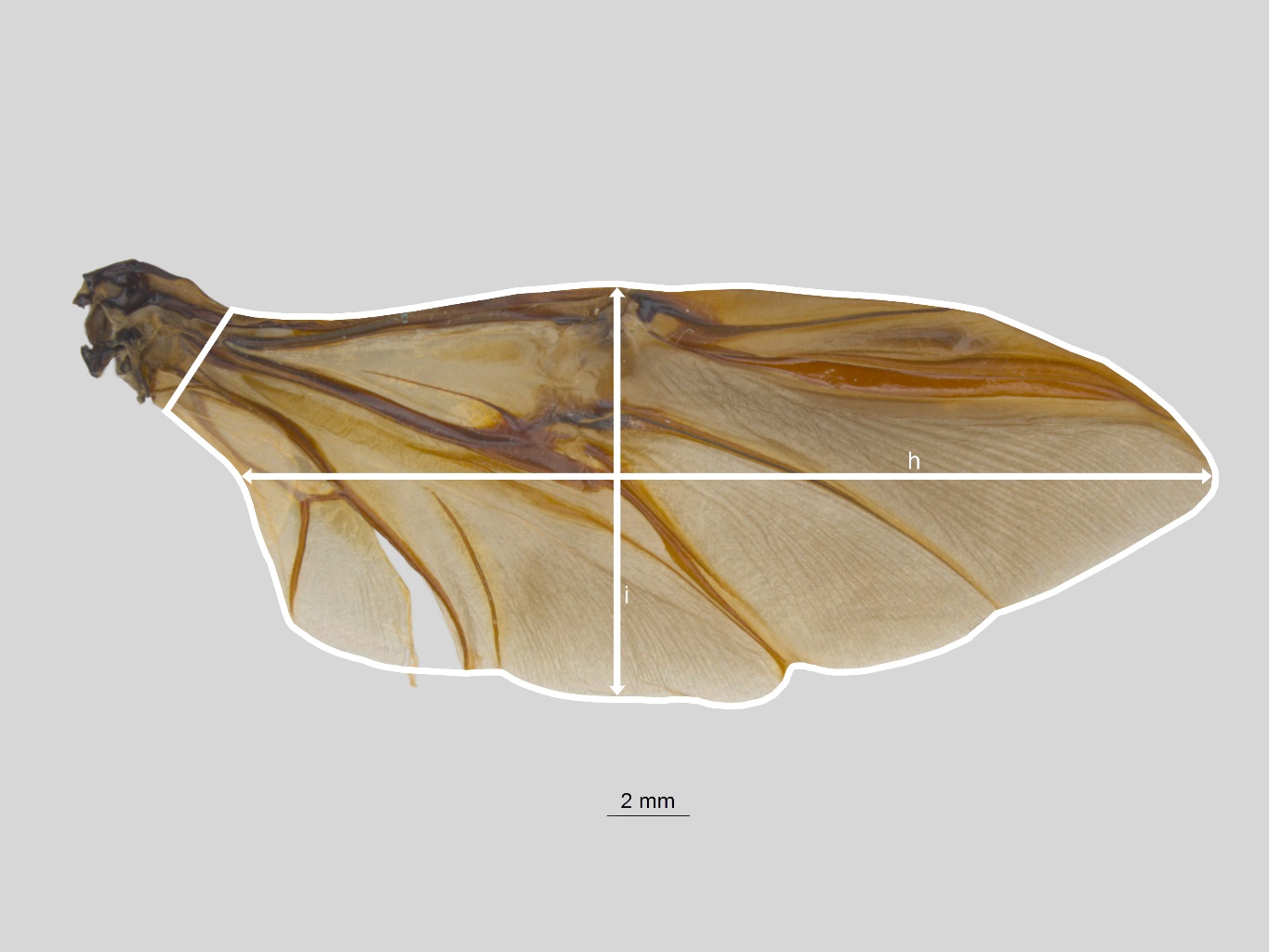


j


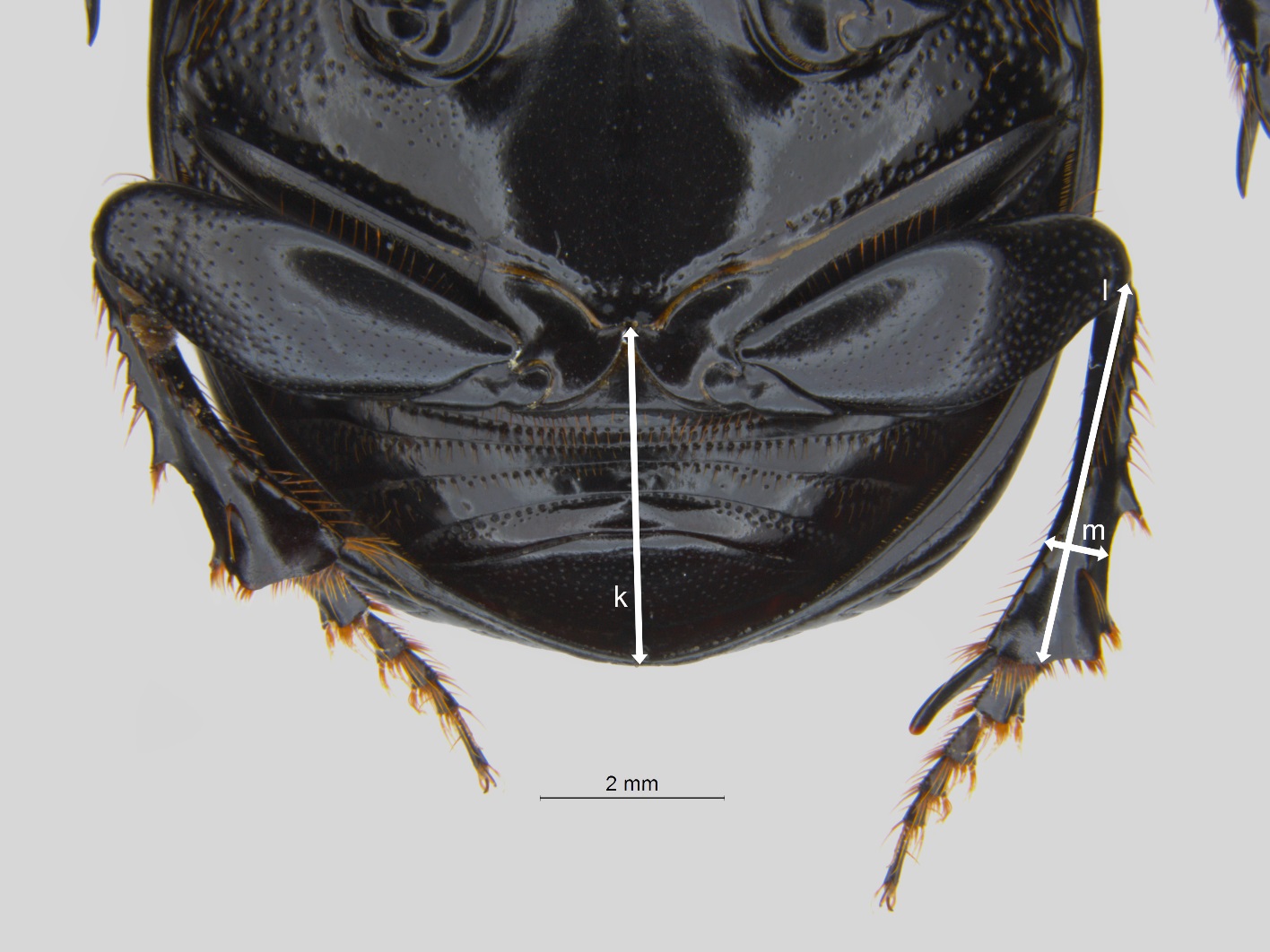


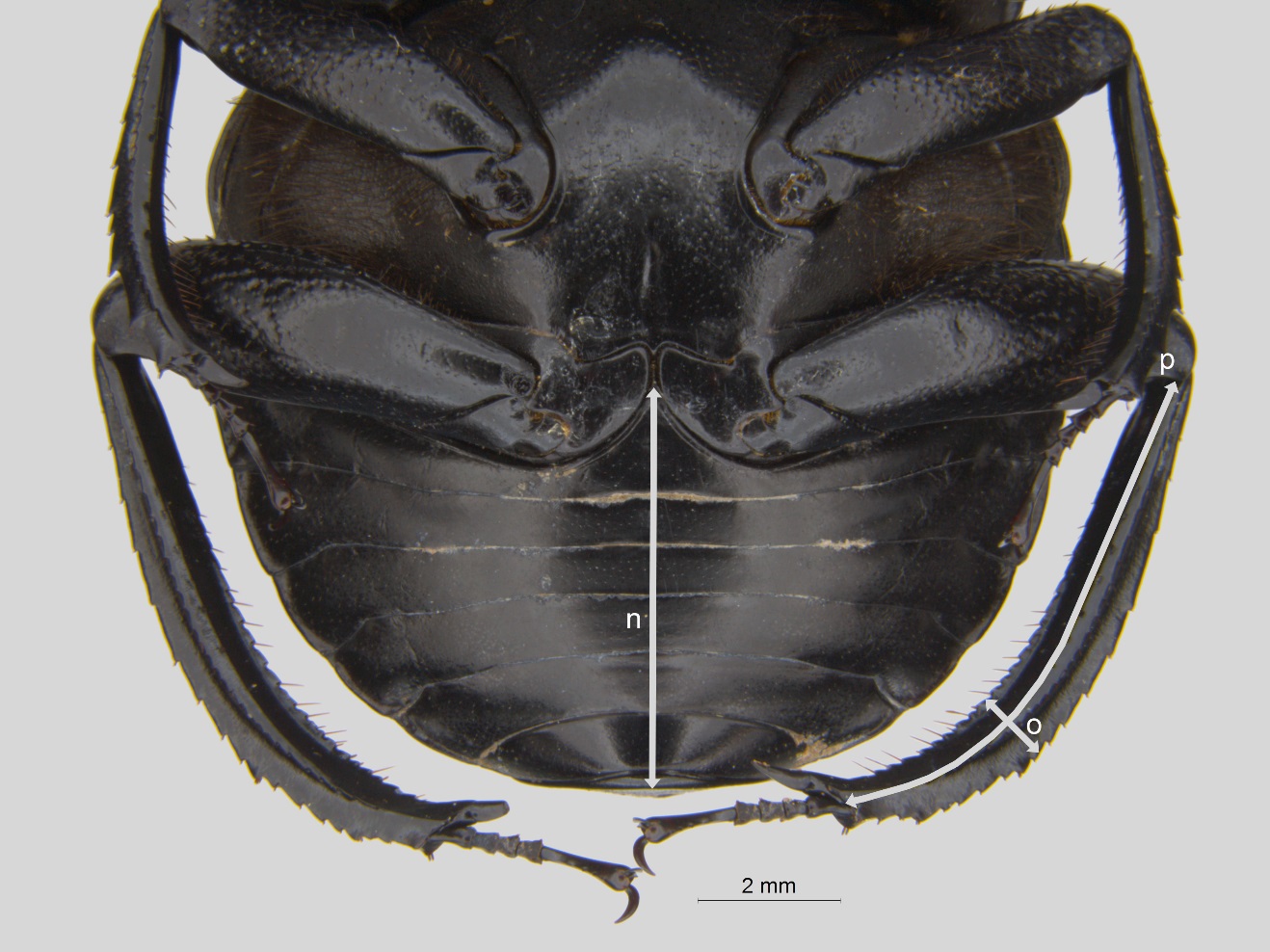


## Figure S1.

Images of *Copris sinicus* showing measurements taken for (a) body length, (b) thorax width, (c) thorax length, (d) head width, (e) eye width, (f) eye length, (g) head length, (h) wing length, (i) wing width, (j) wing area, (k) abdomen length, (l) hind tibia length, (m) hind tibia width. Images of *Paragymnopleurus sparsus* showing measurement taken for (k) abdomen length, (l) alternate hind tibia length (curved) and (m) hind tibia width.

# References (supplementary material)

Barton, P.S., Gibb, H., Manning, A.D., Lindenmayer, D.B. & Cunningham, S. a. (2011) Morphological traits as predictors of diet and microhabitat use in a diverse beetle assemblage. *Biological Journal of the Linnean Society*, **102**, 301–310.

Berwaerts, K., Dyck, H. Van & Aerts, P. (2002) Does flight morphology relate to flight performance? An experimental test with the butterfly Pararge aegeria. *Functional ecology*, **16**, 484–491.

Caveney, S., Scholtz, C. & McIntyre, P. (1995) Patterns of daily flight activity in onitine dung beetles (Scarabaeinae: Onitini). *Oecologia*, **103**, 444–452.

Srygley, R. & Chai, P. (1990) Flight morphology of Neotropical butterflies: palatability and distribution of mass to the thorax and abdomen. *Oecologia*, **84**, 491–499.

Slade, E.M., Mann, D.J. & Lewis, O.T. (2011) Biodiversity and ecosystem function of tropical forest dung beetles under contrasting logging regimes. *Biological Conservation*, **144**, 166–174.
